# Autogenous Chin Block Grafts in the Aesthetic Zone: A 20-Year Follow-Up Case Report

**DOI:** 10.1155/2020/6525797

**Published:** 2020-06-09

**Authors:** Carlo Maiorana, Susanna Ferrario, Pier Paolo Poli, Mattia Manfredini

**Affiliations:** Fondazione IRCCS Ca' Granda Ospedale Maggiore Policlinico, Implant Center for Edentulism and Jawbone Atrophies, Maxillofacial Surgery and Odontostomatology Unit, University of Milan, Via della Commenda 10, 20122 Milan, Italy

## Abstract

The successful use of osseointegrated implants in the treatment of partial or complete edentulism requires a sufficient bone support. Whenever rehabilitation in atrophic edentulous areas is needed, bone augmentation procedures are recommended. The aim is to provide adequate amount of supporting bone to achieve a prosthetically guided implant placement. This in turn leads to functional and aesthetic improvements that can be maintained on the long term. Bone grafting of the atrophic site can be performed either prior to implant placement or at the time of implantation. Irrespective of the timing, bone augmentation by means of autogenous bone grafts is a reliable technique, as confirmed by several studies. On the other hand, long-term evidence on the use of autogenous chin block grafts in preprosthetic implant surgery is still scarce. Thus, the purpose of the present case is to report the 20-year clinical and radiological outcome of autogenous chin block grafts used to augment a bilateral defect due to agenesis of the upper lateral incisors for implant placement purposes.

## 1. Introduction

The agenesis of the upper lateral incisors is a challenging clinical situation, being an area of high aesthetic value with a limited space available for a correct implant insertion, even after an orthodontic treatment, together with high patient expectations [1].

The agenesis often results in alveolar bone defects, especially at the expense of the buccal plate. The repair of congenital or acquired alveolar defects with autologous bone grafts is one of the most traditional surgical techniques in oral and maxillofacial surgery. Once the ideal amount of bone has been restored, implant treatment assumes an important role in the rehabilitation of these patients [2].

Bone reconstruction techniques have been improved in order to optimize the aesthetic and functional result [3, 4]. Despite this, the functional rehabilitation of atrophic alveolar ridges still remains a challenge in oral implantology. Bone augmentation procedures are often indicated to allow implant placement in an optimal three-dimensional position to achieve long-term function and predictable aesthetic results for prosthetic restorations [3]. The extension of the bone defect determines whether bone augmentation procedures can be performed simultaneously with implant placement or as a separate procedure [5].

Among the various bone augmentation materials available, only autologous bone combines osteoconductive, osteoinductive, and osteogenic features when compared to any other bone substitute [6]. Thanks to its biological properties and also due to the absence of immunological reactions, autologous bone graft is considered the “gold standard” for bone regeneration procedures [7].

However, intraoral harvesting is not free from limitations; these include the extension of the donor site and possible complications that may occur during the bone harvesting procedure [8].

The use of intraoral donor sites such as mandibular symphysis and ramus has several advantages compared to that of extraoral sites such as the iliac crest and the tibial plateau. Some studies have shown that membranous bone grafts, including the mandibular symphysis and ramus, show less resorption and better and faster revascularization than endochondral bone grafts, such as the iliac crest and tibial plateau [9, 10]. Therefore, the embryological origin of the donor site plays a pivotal role in the success of the procedure. An additional important advantage of intraoral donor sites is that bone harvesting can be performed with local anesthetic infiltrations without recurring to general anesthesia. Furthermore, intraoral bone grafts can be easily obtained with lower complications compared to extraoral bone grafts, and also, the postoperative course is easier and faster [11]. Common donor sites in the oral region are mandibular symphysis, retromolar area, and maxillary tuberosity [11, 12].

The mandibular symphysis not only provides a greater graft volume of more than 50% compared to the mandibular ramus but is also characterized by a simpler surgical access. Moreover, it has been proved that the mandibular symphysis graft is composed on average of 65% cortical bone and 36% cancellous bone. Conversely, the mandibular ramus is almost 100% cortical in nature. The corticocancellous nature of the bone harvested from the mandibular symphysis facilitates a faster vascularization once the block is positioned at the recipient site, resulting in more rapid integration. Better postoperative patient morbidity and low rate of wound dehiscence are other reasons why the ramus as a donor site may be preferred [11].

The use of bone substitutes characterized by low turnover rates to cover the graft may reduce the resorption rate of the bone block [13]. Some authors found that deproteinized bovine bone (DBB) particles stabilized by resorbable membranes covering onlay block grafts reduced resorption by almost 50% in comparison to noncovered grafts [1, 14]. Bone substitutes may also contribute to the creation of a smooth connection between the block graft and the recipient bone and can provide a scaffold that promotes bone regeneration [13, 14]. Another advantage of using resorbable rather than nonresorbable membranes is the elimination of a second surgical phase.

Long-term studies evaluating the survival rates of dental implants placed in sites augmented with symphysis onlay grafts are lacking. However, to the best of our knowledge, no studies are currently available reporting on survival analysis performed with more than 10 years of follow-up.

In view of the aforesaid, the aim of the present case report was to evaluate the survival rate of dental implants placed in resorbed alveolar ridges reconstructed with symphysis autogenous onlay bone grafts.

## 2. Case Presentation

A 19-year-old male affected by agenesis of the upper lateral incisors was referred to the authors' department seeking for an implant-supported fixed rehabilitation.

At the time of presentation, the patient was healthy, nonsmoking, with no local or systemic pathologies nor drug allergies (ASA I according to the American Society of Anesthesiologists physical status classification).

**Figure 1:**
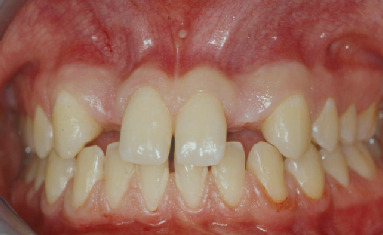
Initial clinical situation.

**Figure 2:**
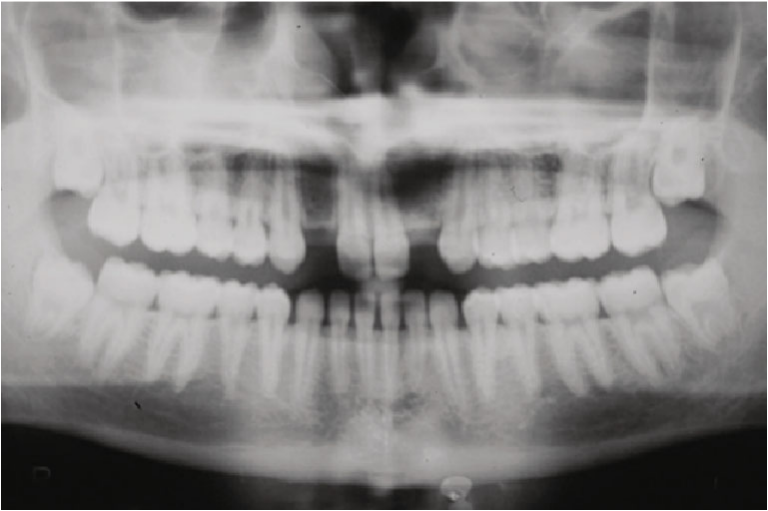
Initial orthopantomography.

The anatomy of the upper jaw was evaluated by clinical examination and panoramic radiograph. At the clinical examination, it was immediately possible to observe a bilateral bone defect in correspondence with the upper lateral incisor region. The appearance and consistency of the soft tissues were good (Figure 1). The orthopantomograph confirmed the agenesis of the upper lateral incisors with a reduced development of the alveolar process in a mesiodistal direction (Figure 2).

After discussing possible treatment alternatives with the patient, it was decided to proceed with a bone augmentation procedure by means of intraoral autogenous bone harvested from the mandibular symphysis and delayed implant insertion. All surgical and prosthetic procedures were performed by the same team. A signed informed consent was obtained from the patient. All procedures were conducted according to the 1964 Helsinki Declaration and its later amendments.

**Figure 3:**
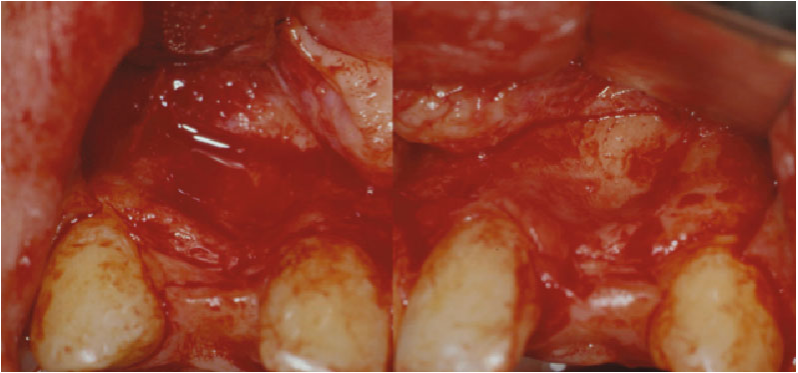
Buccal view of the defects on 12 and 22.

**Figure 4:**
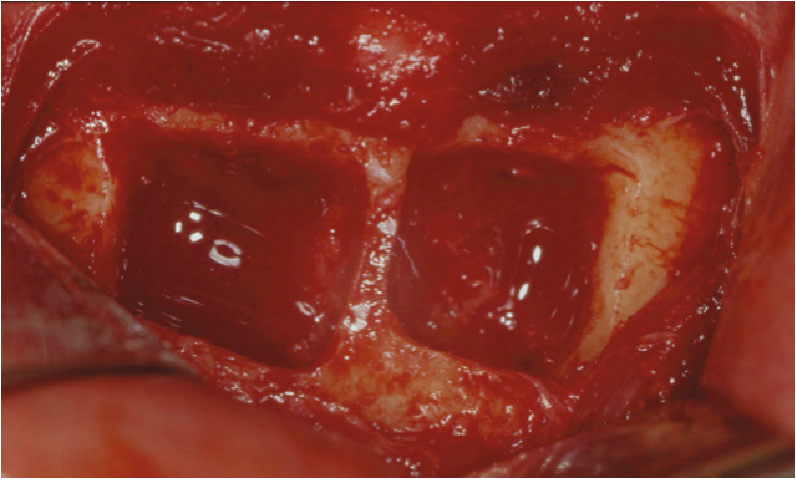
Bone blocks' removal from the mandibular symphysis.

**Figure 5:**
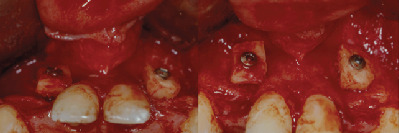
Bone block stabilization by screws.

**Figure 6:**
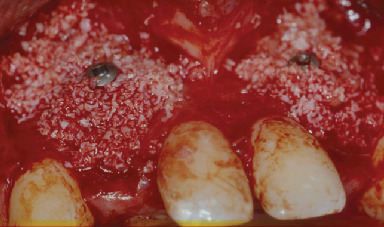
Coverage of the surgical site with deproteinized bovine bone (Bio-Oss®, Geistlich Biomaterials, Wolhusen, Switzerland).

The first surgical phase was performed on an outpatient basis under local anesthesia after premedication with diazepam 0.2 mg/kg administered orally 30 minutes before surgery. Two monocortical block grafts were collected from the symphysis and fixed at the buccal aspect of the bone defects with osteosynthesis screws (Figures 3–5). At this point, DBB particles (Bio-Oss®, Geistlich Biomaterials, Wolhusen, Switzerland) and native lyophilized type I resorbable collagen membranes from equine origin (Paroguide®, GABA VEBAS srl, Rome, Italy) were used to cover the block graft at each site (Figure 6). Suture in polyamide was performed with detached stitches to obtain a first-intention seal of the flaps. Silk sutures were instead used at the donor site.

Postoperative medications included amoxicillin 1 g twice daily for 6 days, starting on the day of surgery, naproxen sodium as required every 6  hours, and 0.2% chlorhexidine mouthwashes twice daily for 2 weeks, starting on the day after surgery.

**Figure 7:**
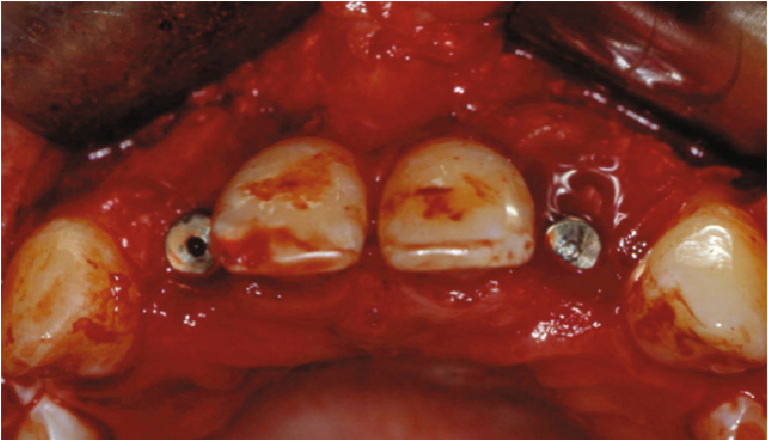
Implant placement.

The sutures were removed after 14 days, and an orthopantomograph was performed. After 6 months of uneventful healing, fixation screws were removed, and two 3.25 × 13 mm implants were placed in a prosthetically guided position with the aid of a surgical stent (Figure 7). The insertion torque was >35 Ncm.

After 5 months, implants were uncovered to connect the healing abutments. After proper maturation of the soft tissues, impressions were taken with custom impression trays to start the prosthetic phase. Temporary implant-supported acrylic resin prostheses were connected to the implants for initial load and soft tissue conditioning. After 6 months, definitive implant-supported cemented-retained metal-ceramic prostheses were delivered. A buccal peri-implant gingival plastic surgery was performed with a diamond bur to improve the aesthetic of the soft tissues.

**Figure 8:**
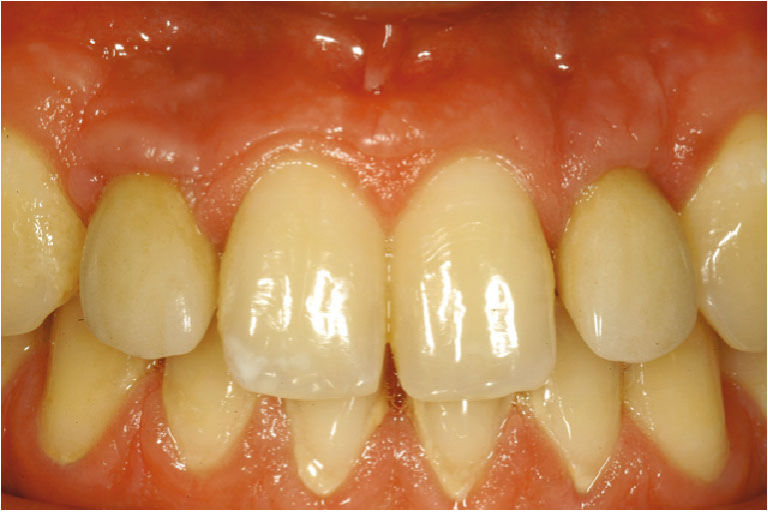
Clinical situation at 8 years.

**Figure 9:**
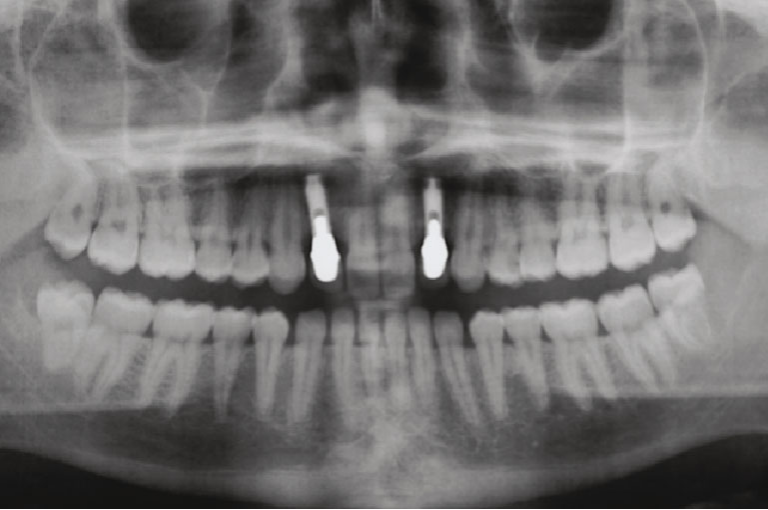
Orthopantomography at 8 years.

**Figure 10:**
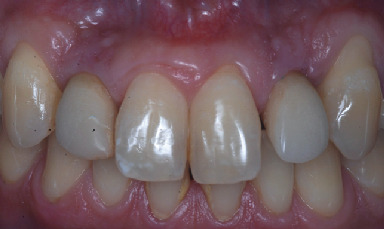
Clinical situation at 20 years.

**Figure 11:**
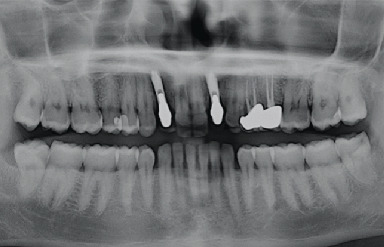
Orthopantomography at 20 years.

Clinical and radiological evaluations were conducted at 8 years (Figures 8 and 9) and 20 years from the prosthetic loading (Figures 10 and 11). After 20 years, the clinical examination showed healthy and stable soft tissues, with no signs of suppuration and bleeding on probing. Peri-implant probing was performed at six sites per implant, namely, mesiobuccal, buccal, distobuccal, mesiopalatal, palatal, and distopalatal. In all said sites, probing depth values ≤ 4 mm were observed. An adequate amount of attached keratinized tissue was present apically to the gingival margin. The quality and stability of the gingival architecture were supported by the radiographic analysis. The 8-year and 20-year orthopantomographs were scanned to obtain digital images with a resolution of 1200 dpi. The digital images were imported in a specialized computer software (ImageJ 1.49v, Research Services Branch, National Institutes of Health, Bethesda, MD, USA). The calibration of the pixel/millimeter ratio was performed on the basis of a known distance, namely, the length of the implants. The 8-year orthopantomograph revealed no detectable marginal bone resorption, calculated as the distance between the most apical bone-to-implant contact visible in the scanned image and the implant-abutment connection level at the mesial and distal aspects (Figure 9). The same measurements were performed in the 20-year orthopantomography (Figure 11); however, it was not possible to obtain reliable data due to metal artifacts. Nonetheless, the clinical findings suggested the presence of stable marginal bone levels circumferentially around the implants.

## 3. Discussion

The present case was reported to document clinically and radiographically the long-term survival of dental implants placed in atrophic alveolar ridges augmented with mandibular symphysis autogenous onlay grafts. The rationale was to provide evidence that implant rehabilitations in bone reconstructed with autogenous mandibular grafts might constitute a reliable treatment option on a long-term basis. This strengthens the current evidence, as only few studies reported on the outcome of such implant-supported rehabilitations for periods longer than 10 years.

In a recent retrospective study [15], it has been claimed that intraoral bone grafts harvested from the mandibular symphysis, mandibular ramus, and maxillary tuberosity provide a good treatment modality for ridge augmentation. In addition, the amount of bone available from these sites is sufficient for anatomical defects extended up to the width of three teeth [16]. Harvesting of retromolar and symphysis bone grafts is particularly recommended in those cases involving multiple tooth reconstruction in the mandible. The surgical access to the symphysis has been described as being easier than that of the mandibular ramus [11]. Both techniques can be performed on an outpatient basis, while harvesting of bone from distant sites is associated with inpatient care and increased costs [17]. Both the harvesting and grafting procedures are usually performed in the same surgical field. The use of autologous bone in the present case showed excellent survival and success rates. The success of the bone augmentation was confirmed by the stability of the marginal bone levels assessed at the mesial and distal aspects of the implants over the years. It is worth mentioning that at the 20-year follow-up visit, a horizontal remodeling of the buccal plate was observed. This however did not affect the implant stability. The horizontal bone resorption that was found is attributable to the embryological nature of the bone that was grafted and the duration of the follow-up [18]. From the aesthetic aspect, the gingival parabolas have been maintained over time, and therefore, the aesthetic success has been preserved on the long term [19]. The present study confirmed the long-term effectiveness of alveolar ridge augmentation and implant placement by means of autogenous bone grafts [20]. This procedure resulted in stable bone conditions with low risk of mucosal recession over an observation period of 20 years.

It should be noted, however, that a similar clinical situation, currently, can be solved through the use of narrow implants and careful soft tissue management. As a matter of fact, at present, soft tissue augmentation techniques have demonstrated good aesthetic results so that more invasive bone augmentation procedures may be avoided [21]. Furthermore, at the time of surgery, the patient was only 19 years old and a different approach with an additional waiting period of 3 years might be contemplated to prevent the intrusion of the peri-implant tissues that may occur in case of premature implant insertion [22]. The patient also refused presurgical orthodontic treatment aimed at optimizing the interdental spaces of the anterior sector.

In a recent paper [18], an average follow-up of 23.9 months was calculated for literature articles on follow-up of patients with implant rehabilitation in augmented bone with autogenous bone grafts. It is therefore clear that the resorption evaluated in this 20-year case report is worthy of note. The hypothesis that bone substitutes could effectively replace the use of autologous bone with its osteoinductive, osteoconductive, and osteogenic properties is still under investigation. On the other hand, various studies have proved benefits and appropriateness of autogenous tissue for an ideal reconstruction of atrophied ridges before implant surgery [23].

Regarding surgical complications, the postoperative morbidity is commonly related to the management of the soft tissues. The most frequent postsurgical complications included flap dehiscences with or without exposure of the grafts or membrane [24]. The peri-implant mucosa needs to be supported by an adequate three-dimensional volume of alveolar bone, including an intact buccal plate of sufficient height and thickness [4, 25, 26]. Deficiency of the buccal bone anatomy has a negative impact on the aesthetic outcome and is therefore considered a critical causative factor for implant complications and failures [4, 26, 27]. In the present case, no soft tissue complications occurred at any stage.

Postoperative morbidity after mandibular bone harvesting procedures was reported to be mainly related to temporary or permanent neural disturbances involving the inferior alveolar nerve and its branches [23]. It is clear that although precise anatomical limits have been defined on the localization of the mandibular incisor canal, in the bone removal from the mandibular symphysis, there is no objective limit below which the probability of having neurosensory alterations is eliminated. This is due to physiological changes in the course of the mandibular incisor canal. It is therefore advisable to subjectively evaluate the feasibility of the technique on a case-by-case basis through orthopantomographs and second-level investigations such as computed tomography, dental scan, and stereolithographic prototypes. No drawbacks related to neurosensory complications were noted in the present case.

A limitation of the study is the marginal bone loss measurement on the mesial and distal aspects owing to 2D imaging. The measurement of buccal and lingual bone loss can only be performed using 3D imaging modalities. However, panoramic radiographs are frequently used in clinical settings for the evaluation of bone peak stability [2, 28].

The results of the present study pointed out that, in case of agenesis of the upper lateral incisors, bone grafting from the mandibular symphysis and delayed implant placement may provide satisfactory functional and aesthetic outcomes on the long term. Despite a certain resorption of the graft that may occur, correct management of the peri-implant soft tissues and the prosthesis is pivotal to maintain the success on the long term. To date, a similar clinical situation can be resolved through the use of narrow implants and careful soft tissue management. Furthermore, at the time of surgery, the patient was 19 years old. To prevent intrusion, we would wait additional 3 years before surgery.
